# Effects of the royal jelly consumption on post-stroke complications in patients with ischemic stroke: results of a randomized controlled trial

**DOI:** 10.3389/fnut.2023.1227414

**Published:** 2024-01-08

**Authors:** Elham Karimi, Arman Arab, Mahdi Sepidarkish, Fariborz Khorvash, Mohammad Saadatnia, Reza Amani

**Affiliations:** ^1^Department of Clinical Nutrition, School of Nutrition and Food Sciences, Isfahan University of Medical Sciences, Isfahan, Iran; ^2^Research Development Center, Arash Women’s Hospital, Tehran University of Medical Sciences, Tehran, Iran; ^3^Division of Sleep Medicine, Harvard Medical School, Boston, MA, United States; ^4^Medical Chronobiology Program, Division of Sleep and Circadian Disorders, Department of Medicine and Neurology, Brigham and Women’s Hospital, Boston, MA, United States; ^5^Social Determinants of Health Research Center, Health Research Institute, Babol University of Medical Sciences, Babol, Iran; ^6^Isfahan Neurosciences Research Center, Alzahra Hospital, Isfahan University of Medical Sciences, Isfahan, Iran

**Keywords:** royal jelly, ischemic stroke, cognitive function, mental health, fatigue

## Abstract

**Aims:**

There is a paucity of evidence regarding the benefit of royal jelly (RJ) on post-stroke complications in patients with ischemic stroke. To address this knowledge gap, this randomized, triple-blind, placebo-controlled clinical trial was carried out to determine the effects of RJ consumption on post-stroke clinical outcomes.

**Methods:**

Of 64 eligible ischemic stroke patients (45–80 years), 32 were randomized to the RJ and 32 to the placebo groups and completed a 12-week intervention. The intervention group was advised to receive 1,000 mg of RJ dragee daily after breakfast. Post-stroke complications including cognition, fatigue, mental health, and appetite, along with serum levels of brain-derived neurotrophic factor (BDNF), and mid-upper arm circumference (MUAC) were assessed in groups pre-and post-intervention.

**Results:**

After 12 weeks of RJ consumption, cognitive function [adjusted mean difference, 4.71; 95% confidence interval (CI), 1.75 to 7.67], serum levels of BDNF (adjusted mean difference, 0.36; 95% CI, 0.05 to 0.67), stress (adjusted mean difference, −3.33; 95% CI, −6.50 to −0.17), and appetite (adjusted mean difference, 1.38; 95% CI, 0.19 to 2.56) were significantly improved. However, the findings for fatigue (adjusted mean difference, −4.32; 95% CI, −10.28 to 1.63), depression (adjusted mean difference, −1.71; 95% CI, −5.58 to 2.16), anxiety (adjusted mean difference, −2.50; 95% CI, −5.50 to 0.49), and MUAC (adjusted mean difference, 0.36; 95% CI, −0.11 to 0.84) were less favorable.

**Conclusion:**

Findings support the benefits of RJ consumption in improving post-stroke complications and clinical outcomes.

**Clinical trial registration**: https://www.irct.ir/trial/59275, Identifier IRCT20180818040827N4.

## 1 Introduction

Ischemic stroke is considered the second leading cause of disability and death, globally, with a staggering burden on both levels of individual and societal (1). In 2016, it was estimated that 80.1 million people worldwide suffered from ischemic stroke which translated into a global prevalence of ≈1,322 per 100,000 persons (2). Ischemic stroke is higher in some ethnic or racial groups, less educated populations, older women, and those residing in middle- or lower-income countries (3).

In addition to the identification of etiology and risk factors, the post-stroke long-term prognosis is of great interest from the perspective of primary care physicians (4). Late medical consequences and complications of stroke typically occur within weeks to months following stroke including post-stroke seizures, urinary incontinence, bowel incontinence, cognitive impairment, fatigue, mood/emotional changes, muscle wasting, and loss of appetite (4–7).

Accordingly, post-stroke cognitive impairment nearly occurs in over 80% of patients contributing to post-stroke disability (8, 9). Moreover, mood disorders are much more prevalent in stroke patients compared to stroke-free individuals negatively affects cognitive function (8, 9). Likewise, post-stroke depression was shown to increase long-term disability by 15% in ischemic stroke survivors (10). Furthermore, post-stroke fatigue has been linked with the deterioration of several aspects of daily life and also exerts a negative effect on recovery and survival with a prevalence rate ranging from 29% to 77% (11). In addition, malnutrition is observed in up to 50% of patients after stroke predicting post-stroke functional outcomes and complications (12). Agents affecting these long-term complications are believed to diminish the burden of the disease and improve the quality of life of individuals after ischemic stroke.

Royal jelly (RJ) is a white-yellowish secretion of the hypopharyngeal and mandibular glands of young worker bees with a sour taste and smell of phenol (13, 14). It is an acid colloid, with a pH of 3.6 to 4.2, composed mainly of water (60%–70%), carbohydrates (11%–23%), proteins (9%–18%), lipids (4%–8%), vitamins, minerals, and other components (0.8–3%) (14, 15). RJ is a widely used dietary supplement and functional food, which has many biological properties, such as antihypertensive (16), anti-aging (17), anti-allergic (18), antibacterial (19), anti-inflammatory (20), anti-oxidant (21), and antitumor (22). In animal models, oral ingestion of RJ improves cognitive function via the regeneration of hippocampal granule cells (23, 24). Accordingly, RJ consumption in patients with cancer undergoing chemotherapy was effective regarding fatigue and anorexia amelioration (25–27). Likewise, its consumption by postmenopausal women improves mood status including anxiety and depression (28). Evidence may imply the potential role of RJ in individuals with ischemic stroke; however, we were unable to find published clinical trials investigating the beneficial effects of RJ on post-stroke complications and consequences. Given that RJ supplementation is considered generally safe if proven to have a beneficial effect on post-stroke complications, it may offer a supplemental or alternative therapy to the current therapeutic approaches that often have side effects. To address this knowledge gap, we did the current study to investigate the effects of RJ supplementation on post-stroke complications in those with ischemic stroke.

## 2 Methods

### 2.1 Study design

This was a randomized triple-blind placebo-controlled parallel clinical trial investigating the beneficial effects of RJ consumption for 12 weeks among patients with ischemic stroke.

### 2.2 Ethics

The study protocol of the current investigation was published elsewhere with details on design, objectives, and endpoints (29). The fundamentals of the current study were approved by the Medical Research Ethics Committee at the Isfahan University of Medical Sciences (Approval number: IR.MUI.RESEARCH.REC.1400.291; Approval date: 09 October 2021). Then, it was registered at the Iranian Registry of Clinical Trials (Registration number: IRCT20180818040827N4; Registration date: 16 October 2021). Participation was completely voluntary and an informed written consent form was obtained from each patient prior to enrollment. The current study was designed and done following the Declaration of Helsinki (30). Moreover, it was reported based on the Consolidated Standards of Reporting Trials (CONSORT) guidelines (31).

### 2.3 Participants and study settings

From November 2021 to December 2022 patients who were admitted to the Alzahra Hospital, an educational hospital of the Isfahan University of Medical Sciences, with suspected ischemic stroke were evaluated for possibility of enrollment. Patients with a confirmed diagnosis of ischemic stroke, National Institutes of Health Stroke Scale (NIHSS) score of 5–20, and 45–80 years were enrolled in the current trial. Patients were excluded if they (1) had an allergy to honey or its by-products, dermatitis, asthma, acute kidney or liver disease, cardiovascular disease, malignancies, and/or history of stroke with a score of modified Rankin Scale (mRS) ≥ 1; (2) took multivitamins or antioxidants supplements or adhered to any specific diet over the past 12 weeks prior to enrollment; and (3) were pregnant or lactating. Likewise, participants with any adverse reactions to RJ supplements, recurrent stroke, death, or low compliance (consumption of less than 80% of RJ supplements) were also excluded.

### 2.4 Sample size

We calculated the sample size based on the primary outcome, the mRS. We assumed a maximum type 1 error of 0.05 and 80% statistical power, mRS standard deviation equal to 0.8, and an effect size equal to 0.6; a total number of 32 patients were estimated to be enrolled for each group taking into account the 10% dropout factor (32). The mRS is used to measure the degree of disability or dependence on the daily activities of those who have had a stroke (33).

### 2.5 Randomization and blinding

A total of 256 individuals with a suspicious ischemic stroke were admitted to the Alzahra hospital, of which 64 patients who met eligibility criteria were randomly allocated to the RJ (*n* = 32) or placebo (*n* = 32) group in a ratio of 1:1. Randomization was done using permuted block (block size six) and computer-generated random numbers. Randomization codes (six-digit numbers) were provided by an independent statistician in opaque and sealed envelopes and opened sequentially upon patient enrollment. Both RJ and placebo were also coded using the same randomization codes by the manufacturer. Participants, researchers, statisticians, and all who were in contact with patients were unaware of the allocated treatment.

### 2.6 Protocols of the intervention in the experimental and the control groups

Both RJ and placebo were manufactured by Kooze-asal Arya Ravis knowledge-based company (Isfahan, Iran), and were identical in color, shape, flavor, and size. Patients in the intervention (*n* = 32) and the control (*n* = 32) groups were asked to consume RJ and placebo for 12 weeks, respectively. Each RJ dragee contained 30% of RJ powder [21 mg of 10-HDA (10-hydroxy-2-decenoic acid), which corresponds to 1,000 mg of fresh RJ], 53% of honey powder, and 17% of filler. Each placebo dragee contained 53% of honey powder and 47% of filler. Patients were instructed to consume RJ dragee after breakfast and return dragee containers after 12 weeks to improve their compliance. Moreover, they were in direct contact with the first investigator (E.K.). Likewise, they were advised not to change their lifestyle habits in terms of diet and physical activity.

### 2.7 Baseline evaluation

At baseline, each patient was assessed by a neurologist (F.K.) and a nutritionist (E.K.) to obtain relevant information. The severity of the stroke was also evaluated at baseline by the neurologist utilizing the NIHSS tool (34). It is an 11-item instrument assessing extinction/inattention, dysarthria, ataxia, motor arm and leg, gaze, level of consciousness, visual fields, facial palsy, sensory, and language with an overall score of 0–42 (35). Information on medication, age, gender, education, smoking, history of stroke, and time since stroke was gathered through a face-to-face interview. A fasting serum sample was collected to measure C-reactive protein (CRP) at baseline via an immunoturbidimetric method via commercial kits (biorexfars, Shiraz, Iran). The patient’s quality of life was evaluated via the stroke-specific quality of life scale (SS-QOL) through a face-to-face interview at baseline (36, 37).

### 2.8 Dietary intakes

The dietary intake of patients throughout the study was estimated by a 3-day food record questionnaire (2 weekdays and 1 weakened). Participants were instructed to fill out food log sheets at baseline, 6, and 12 weeks at the time they consumed food to diminish their reliance on memory. All food logs were summed up to calculate the overall intake during the study. Collected data were analyzed using the Nutritionist 4 software (First Databank, Hearst Corp, San Bruno, CA, United States).

### 2.9 Study endpoints

All study endpoints were examined at baseline and after 12 weeks of intervention. The primary outcomes were cognitive function, fatigue, and serum levels of brain-derived neurotrophic factor (BDNF). Secondary outcomes were mental health measures (i.e., anxiety, depression, stress), appetite, and mid-upper arm circumference (MUAC).

#### 2.9.1 Cognitive function

Cognitive function was examined by the neurologist through a face-to-face interview via the mini-mental state examination (MMSE) test (38). It is a 30-point questionnaire assessing orientation, attention, language, visual–spatial skills, and memory with an overall score ranging from 0 to 30. Higher values are interpreted as better cognitive function (39).

#### 2.9.2 Fatigue

A face-to-face interview was done by the neurologist to obtain information on fatigue using a validated fatigue severity scale (FSS) questionnaire (40). FSS is a nine-item questionnaire with options ranging from 1 (strong disagreement) to 7 (strong agreement). The overall score would be between 9 and 63 with a higher score representing more severe fatigue (41).

#### 2.9.3 BDNF

A venous blood sample was taken after 12 h of fasting in the clinical laboratory of Alzahra Hospital. The serum level of BDNF was measured using the ELISA method (ZelBio kit, Germany). Serum was extracted by centrifuging blood at 3,500 rpm and then serum was isolated and feezed at −80°C.

#### 2.9.4 Mental health

The mental health of the participants including depression, anxiety, and stress was examined utilizing a validated version of the depression, anxiety, stress scale (DASS-21) questionnaire by the neurologist through a face-to-face interview (42). Each subscale of the DASS-21 questionnaire contains 7 questions scoring from 0 to 3. Then, the overall score should be multiplied by 2 to re-scale the DASS-21 to the original DASS-42. The calculated score can range from 0 to 42 and higher points are indicative of severe mental health status (43).

#### 2.9.5 Appetite

A validated version of the simplified nutritional appetite questionnaire (SNAQ) was completed for each patient by a nutritionist (E.K.) through a face-to-face interview (44). This is a 4-item screening tool with five options for each and an overall score of 4–20. A lower score predicts the risk of weight loss in the next 6 months (45).

#### 2.9.6 MUAC

MUAC was measured using a non-stretchable tape to the nearest 0.1 cm by the first investigator (E.K.). It represents the arm circumference in the midpoint between the acromion on the shoulder blade and the olecranon processes of the ulna. This value denotes average amounts of subcutaneous fat and muscle in the upper arm (46).

### 2.10 Statistical analysis

We described the continuous and categorical variables by mean (SD) and counts (percentages), respectively. Baseline characteristics were compared between the two groups using the independent sample t-test for continuous data and a Chi-squared test for categorical data. The continuous primary and secondary endpoints were analyzed by a multivariable mixed-effect linear model. In this model, the baseline measurement of an endpoint and potential confounders was controlled. During the trial, nine subjects (four in RJ and five in the placebo groups) failed to complete the final measurements, resulting in some incomplete observations. These incomplete observations were imputed using multiple imputations based on chained equations, which filled in missing values in multiple variables iteratively using a sequence of univariate imputation models with a fully conditional specification of prediction equations. The estimated treatment differences from the multivariable model were, therefore, reported in adjusted mean differences with corresponding 95% confidence intervals (CI). The reported value of p was two-sided and a value of <0.05 was considered statistically significant. All statistical analyses were performed by Stata software 17 (Stata Corp, College Station, TX, United States).

## 3 Results

**Figure 1:**
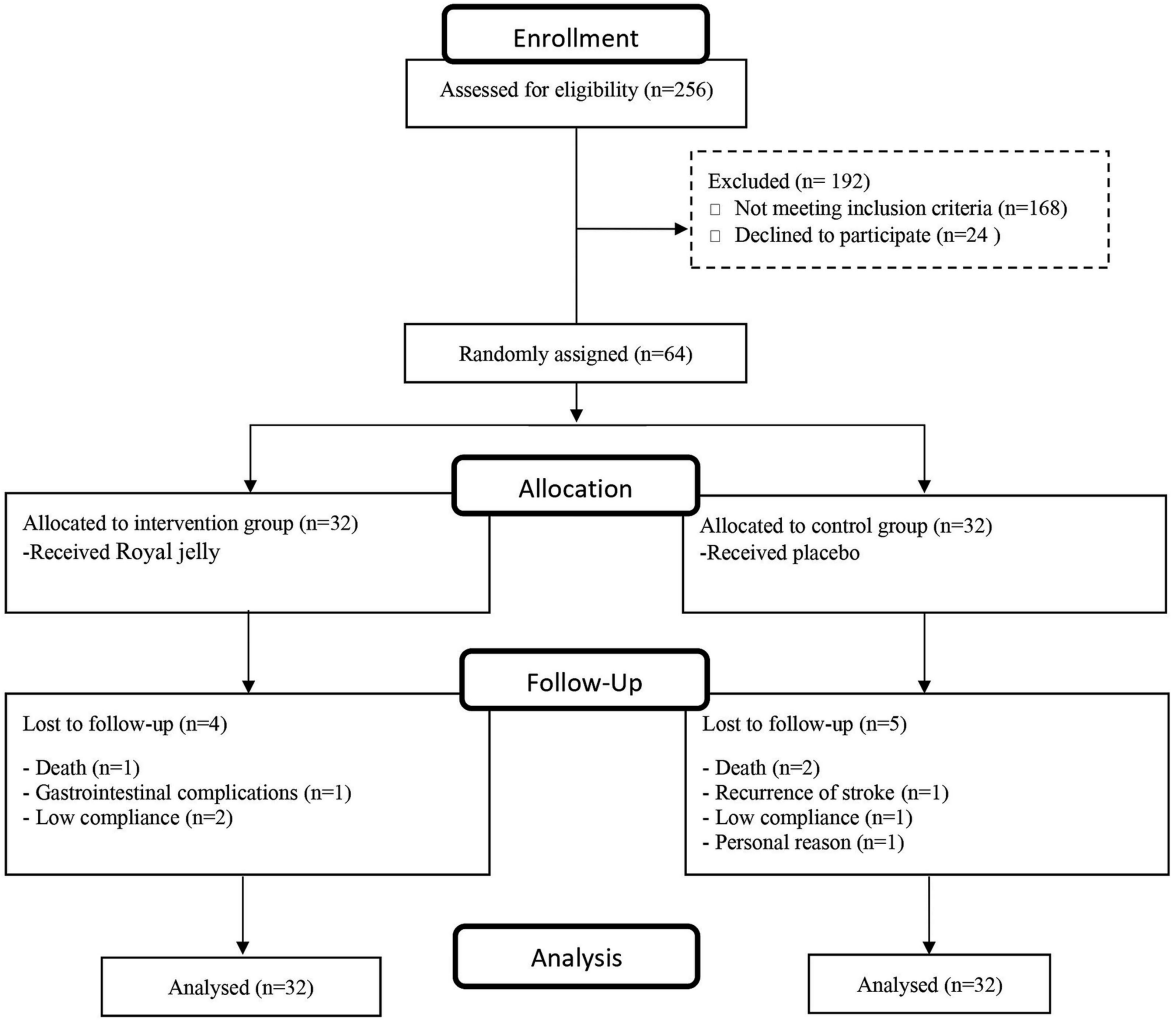
CONSORT flow diagram of study participants.

The trial profile of the current study is shown in Figure 1. Of 256 individuals who were assessed for eligibility, 64 patients with ischemic stroke met our inclusion criteria and were randomly assigned to receive RJ or a placebo. After 12 weeks of intervention, four patients in the intervention group [due to low compliance (*n* = 2), gastrointestinal complications (*n* = 1), and death (*n* = 1)] and five in the placebo group [due to low compliance (*n* = 1), personal reason (*n* = 1), recurrent stroke (*n* = 1), and death (*n* = 2)] were lost to follow up. An intention-to-treat (ITT) technique was implemented to analyze the data and hence all randomized patients were considered for final analysis. No unfavorable event was observed during 12 weeks of RJ consumption.

**Figure 2:**
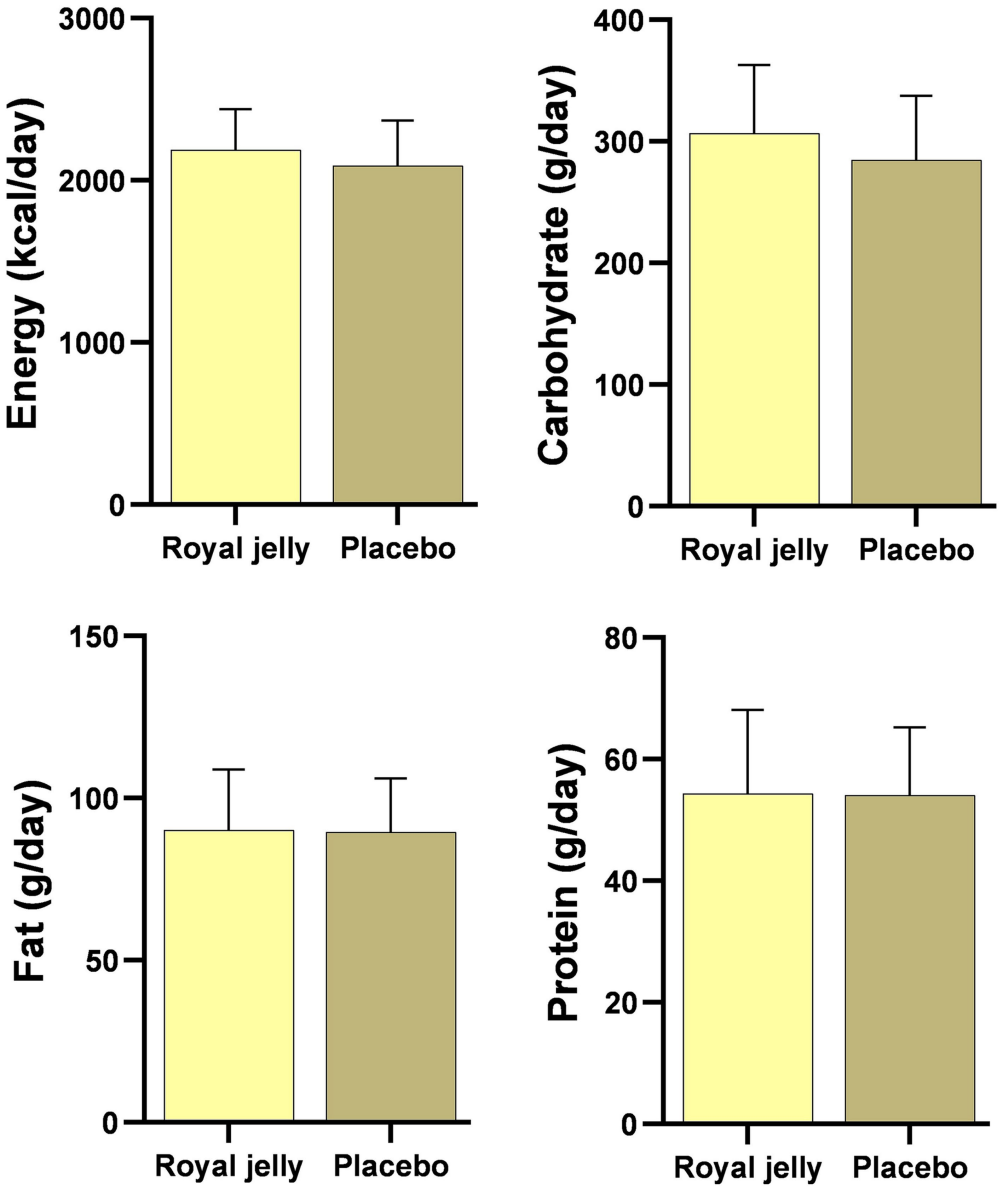
Dietary intakes of the intervention group (royal jelly = 32) and the control group (placebo = 32) during the three-month intervention. Nutrient intakes are presented as means with standard deviation within bars.

The baseline characteristics of participants randomly assigned to treatment or control group are presented in Table 1. Clinical and demographic characteristics of the patients with ischemic stroke at randomization were comparable between the intervention and control groups. Similarly, energy, protein, fat, and carbohydrate were comparable between the groups during 3 months of intervention (Figure 2).

**Table 1:** Baseline characteristics of the study population.

	Royal jelly (*n* = 32)	Placebo (*n* = 32)	*p*-value
Age (year)	65.56 ± 11.54	65.03 ± 10.84	0.850
Female	15 (46.9)	17 (53.1)	0.617
Diploma or lower education	24 (75.0)	18 (56.3)	0.058
Current smoker	10 (31.3)	4 (12.5)	0.070
History of stroke	8 (25.0)	6 (18.8)	0.424
Time since stroke	4.68 ± 2.40	5.84 ± 4.94	0.239
NIHSS	8.09 ± 3.20	9.46 ± 4.25	0.149
CRP (mg/L)	18.71 ± 28.64	16.28 ± 27.53	0.730
Quality of life	138.37 ± 47.12	131.21 ± 42.87	0.528
**Medications**			
Antihyperlipidemic	18 (56.3)	25 (78.1)	0.062
Antihyperglycemic	12 (37.5)	9 (28.1)	0.424
Anticoagulant	27 (84.4)	26 (81.3)	0.740
Antihypertensive	22 (68.8)	25 (78.1)	0.396

Data are presented as mean ± SD or number %. NIHSS, National Institutes of Health Stroke Scale; CRP, C-Reactive Protein.

After 12 weeks of RJ consumption, the MMSE score was higher in the intervention group compared to the control group (17.16 v. 11.62) with an adjusted mean difference of 4.71 (95% CI, 1.75 to 7.67). The fatigue score did not differ between the RJ and the placebo groups (adjusted mean difference, −4.32; 95% CI, −10.28 to 1.63). Serum levels of BDNF increased significantly following 12 weeks of RJ supplementation with an adjusted mean difference of 0.36 (95% CI, 0.05 to 0.67). In the end, patients in the RJ group had significantly lower stress scores than those of the placebo group (adjusted mean difference, −3.33; 95% CI, −6.50 to −0.17). However, the beneficial effects of RJ consumption were not observed for other domains of mental health including depression and anxiety. After adjustment for baseline values of appetite, CRP, age, smoking, NIHSS, history of stroke, fatigue, stress, anxiety, and depression, the adjusted mean difference for appetite was 1.38 (95% CI, 0.19 to 2.56) for patients who received RJ compared to those of the placebo group. The MUAC did not differ between the groups (adjusted mean difference, 0.36; 95% CI, −0.11 to 0.84). The findings of the post-stroke complications and consequences are indicated in Table 2.

**Table 2:** Ischemic stroke complications pre- and post-intervention.

		Intervention (*n* = 32)	Control (*n* = 32)	Mean difference (95% CI)	*p-*value
MMSE	Pre	14.12 ± 8.25	12.53 ± 9.23	1.59 (−2.78, 5.97)	0.469
	Post	18.01 ± 8.29	13.44 ± 8.58	**4.71 (1.75, 7.67)** ^a^	0.002^*^
Fatigue	Pre	34.06 ± 14.70	33.96 ± 13.67	0.09 (−7.00, 7.19)	0.979
	Post	34.89 ± 13.20	39.84 ± 14.86	**−4.32 (−10.28, 1.63)** ^**a**^	0.154^*^
BDNF (ng/mL)	Pre	3.00 ± 0.48	2.78 ± 0.56	0.22 (−0.03, 0.48)	0.093
	Post	3.45 ± 0.60	3.12 ± 0.62	**0.36 (0.05, 0.67)** ^b^	0.022^*^
Depression	Pre	16.56 ± 11.65	16.18 ± 11.39	0.37 (−5.38, 6.13)	0.897
	Post	20.86 ± 11.43	17.83 ± 10.39	**−1.71 (−5.58, 2.16)** ^**c**^	0.383^*^
Anxiety	Pre	12.87 ± 9.10	12.53 ± 9.98	0.34 (−4.42, 5.11)	0.886
	Post	11.81 ± 8.36	10.15 ± 10.13	**−2.50 (−5.50, 0.49)** ^**c**^	0.101^*^
Stress	Pre	20.71 ± 10.86	21.64 ± 12.09	0.43 (−5.14, 6.02)	0.876
	Post	20.14 ± 9.50	24.57 ± 12.12	**−3.33 (−6.50, −0.17)** ^**c**^	0.039^*^
Appetite	Pre	16.12 ± 2.72	16.15 ± 2.61	−0.03 (−1.36, 1.30)	0.963
	Post	17.03 ± 2.62	16.23 ± 2.98	**1.38 (0.19, 2.56)** ^**a**^	0.023^*^
MUAC (cm)	Pre	29.68 ± 2.44	29.00 ± 2.18	0.68 (−0.47, 1.84)	0.241
	Post	28.88 ± 2.48	28.07 ± 1.70	**0.36 (−0.11, 0.84)** ^**d**^	0.136^*^

Data are presented as mean ± SD. Bold values are presented as adjusted mean difference (95% CI). ^*^Calculated by mixed-effect linear model. ^a^Adjusted for baseline values, age, sex, education, history of stroke, times since stroke, NIHSS and quality of life. ^b^Adjusted for baseline values, age, smoking, history of stroke, NIHSS, CRP, stress, anxiety, depression and fatigue. ^c^Adjusted for baseline values, age, sex, education, times since stroke, NIHSS and quality of life. ^d^Adjusted for baseline values, age, sex, NIHSS and antihyperlipidemic agent. MMSE, Mini-Mental Examination Exam; BDNF, Brain Derived Neurotrophic Factor; MUAC, Mid-Upper Arm Circumference; NIHSS, National Institutes of Health Stroke Scale; CRP, C-reactive protein.

## 4 Discussion

This trial was designed to address the knowledge gap regarding the beneficial role of RJ consumption in improving post-stroke complications and consequences in patients with ischemic stroke. Previous trials were limited on this topic and our findings can extend the existing literature regarding the potential role of RJ; however, our findings should be interpreted with caution owing to the preliminary nature of our investigation. We found that RJ administration for 12 weeks in individuals with ischemic stroke was beneficial in improving cognitive function, serum levels of BDNF, stress, and appetite. However, it may be less favorable regarding fatigue, depression, anxiety, and MUAC.

We observed an increase of 21.5% in the score of MMSE in the RJ group compared to the 7.26% reduction in the placebo group. A previous pooled analysis reported a minimum clinically important difference (MCID) of 1.4 points for MMSE in patients with dementia which was lower than our observed adjusted mean difference (4.71) and consequently may confirm the clinical importance of our results (47). Our study confirmed previous reports regarding the potential efficacy of RJ in improving cognitive function in animal models (24, 48–52). In a single clinical trial, 66 individuals with mild cognitive impairment (MCI) consumed a daily capsule of Memo®, a triple combination of *Panax ginseng* (150 mg), *Ginkgo biloba* (120 mg), and 750 mg of lyophilized RJ, for 30 days. MMSE was significantly improved following the administration of Memo® compared to the placebo (53). RJ may improve cognition through various pathways with a network of interrelated mechanisms (54). RJ activates AMP-activated protein kinase (AMPK) and leads to the suppression of microglial inflammation via inhibition of various apoptotic, inflammatory, and oxidative pathways, e.g., nuclear factor-kappa B (NF-κB), and inducible nitric oxide synthase (iNOS) (54). Moreover, RJ consumption enhances the production of neurotrophins such as BDNF and nerve growth factor (NGF) promoting synaptogenesis, neurogenesis, and acetylcholine production (54).

Accordingly, we found a significant increase in the serum levels of BDNF after adjustment for potential confounders. This observation may in part justify an improvement of cognitive function after RJ consumption since BDNF regulates the proliferation of dendritic arbor, axonal sprouting, and synaptic plasticity (55). 10-HDA-related esters of RJ are reported to produce intracellular BDNF-like signals and also upregulate the mRNA expression of BDNF (56–58). Eventually, research using RJ to increase serum levels of BDNF in humans is scarce and therefore further attempts are needed.

We failed to observe a significant improvement in the score of FSS after 12 weeks of RJ supplementation. In contrast to our findings, RJ was reported to be beneficial in diminishing cancer-related fatigue after 3 months of intervention (25–27). This discrepancy could be attributed to the difference in the pathophysiology of cancer-related fatigue compared to post-stroke fatigue (59, 60). Post-stroke fatigue could be linked with type of stroke, stroke lesion location, white matter lesions and brain atrophy, hypothalamus-pituitary axis dysregulation, neurotransmitter changes, and/or inflammation (59, 61–63). However, cancer-related fatigue is mostly multi-factorial and includes inter-related neuroendocrine, neurotransmitter, muscular, and cytokine changes (64). We found a 4.32-point decrease in the score of FSS after RJ consumption without reaching significant levels. Hence, other studies with larger sample sizes, longer duration, and/or higher doses of RJ may intensify the magnitude of our observed effect size.

RJ consumption led to an improvement in the score of stress together with less favorable findings for depression and anxiety. RJ was shown to possess a mood-enhancing potential in animal models (65–68). A single clinical trial demonstrated that 800 mg of RJ consumption for 12 weeks improves anxiety in post-menopausal women (28). In contrast, other evidence failed to provide a beneficial effect of RJ in improving mood status in asymptomatic overweight adults (69). The therapeutic potential of RJ for psychological disorders is associated with various activities, e.g., binding with estrogen receptors to modulate cell proliferation, counteracting neuroinflammation, scavenging free radicals, and mimicking the effect of BDNF (57).

Post-stroke appetite improved after 12 weeks of RJ consumption by 5.64% compared to 0.49% in the placebo group. Subcutaneous injection of freeze-dried RJ in 8 cases of malnourished infants proposed promising results (70). Moreover, RJ was effective in improving appetite in patients with malignancy (71, 72) together with another contradictory finding among overweight adults (69). The observed increase in post-stroke appetite was accompanied by a tendency to higher macronutrient intakes including total energy, carbohydrate, protein, and total fat in the RJ group compared to the placebo group; however, the identified difference was not statistically significant. Therefore, it might be assumed that with a larger sample size, we can observe significant results for the dietary intake of patients. Following this observation, 8 weeks of RJ consumption failed to alter the dietary intakes of overweight adults (69). However, RJ consumption reduces the dietary intake of energy and carbohydrates in patients with type 2 diabetes mellitus (73). Differences in the type of study population might be the main reason for the observed discrepancy in findings. Inflammation and oxidative stress play a role in post-stroke loss of appetite, as well as in the cascade of ischemic events in the brain area. Therefore, reducing oxidative stress and inflammation subsequently leads to an improvement in the appetite and dietary intake of patients with stroke (74).

Furthermore, although MUAC increased in the RJ group after controlling for potential confounders, it was not statistically significant. Since there was no significant difference between the two groups regarding 24-h energy intake, it might justify the result for MUAC, and with a higher dose of RJ or supplementation for a longer duration, we may observe a more favorable outcome for MUAC. The findings of previous reports on the link between RJ and body composition are contradictory. RJ consumption reduced body weight in diabetic patients which was suggested to be linked with the upregulation of oxidative phosphorylation and oxygen metabolism (73). On the other hand, consumption of 6 g/day of RJ did not alter waist circumference, fat mass, and body weight in healthy individuals (75). Moreover, 8 weeks of RJ consumption failed to show any substantial changes in the level of body fat and fat-free mass in overweight adults (69).

To the best of our knowledge, this is the first randomized trial investigating the beneficial role of RJ in patients with ischemic stroke. Moreover, we used a triple-blind approach adjusted for potential confounders to control for possible sources of bias. However, some limitations warrant consideration. Patients were recruited in a single center and, therefore, multi-center studies elaborate generalizability of the findings. Furthermore, our findings should be considered as a preliminary step, and further investigation is required. The sample size was relatively small and future attempts should investigate the role of RJ in a larger study population. In our study, patients consumed 1,000 mg RJ and hence further trials with different doses can elaborate our understanding of possible dose-dependent effects of RJ.

## 5 Conclusion

RJ supplementation for 12 weeks in patients with ischemic stroke seems to be beneficial in terms of cognitive function, serum levels of BDNF, stress, and appetite. However, no significant changes were observed for fatigue, depression, anxiety, and MUAC. Considering that our results are preliminary, further trials are warranted to confirm our findings.

## Data availability statement

The raw data supporting the conclusions of this article will be made available by the authors, without undue reservation.

## Ethics statement

The current study protocol was approved by the Research Ethics Committee at the Isfahan University of Medical Sciences on 9 October 2021 (IR.MUI.RESEARCH.REC.1400.291) and was registered at the Iranian Registry of Clinical Trials on 16 October 2021 (IRCT20180818040827N4). The studies were conducted in accordance with the local legislation and institutional requirements. Written informed consent for participation in this study was provided by the participants’ legal guardians/next of kin.

## Author contributions

EK, AA, and RA: conception and design. EK, FK, and RA: acquisition of data. AA and MaS: analysis and interpretation of data. EK and AA: drafting the manuscript. AA, FK, MS, EK, MaS, and RA: revising it for intellectual content. All authors contributed to the article and approved the submitted version.
